# Vascular endothelial growth factor levels in diabetic peripheral neuropathy: a systematic review and meta-analysis

**DOI:** 10.3389/fendo.2023.1169405

**Published:** 2023-05-12

**Authors:** Rui Ding, Shicong Zhu, Xiaoyan Zhao, Rensong Yue

**Affiliations:** ^1^ Department of Endocrinology, Hospital of Chengdu University of Traditional Chinese Medicine, Chengdu, China; ^2^ Department of Respiratory, Hospital of Chengdu University of Traditional Chinese Medicine, Chengdu, China

**Keywords:** diabetic peripheral neuropathy, vascular endothelial growth factor, angiogenesis, microcirculation, meta analysis

## Abstract

**Objective:**

Vascular endothelial growth factors (VEGFs, including VEGF-A, VEGF-B, VEGF-C, VEGF-D and PLGF) have important roles in the development and function of the peripheral nervous system. Studies have confirmed that VEGFs, especially VEGF-A (so called VEGF) may be associated with the diabetic peripheral neuropathy (DPN) process. However, different studies have shown inconsistent levels of VEGFs in DPN patients. Therefore, we conducted this meta-analysis to evaluate the relationship between cycling levels of VEGFs and DPN.

**Methods:**

This study searched 7 databases, including PubMed, Embase, Cochrane Library, China National Knowledge Infrastructure (CNKI), VIP Database, WanFang Database, and Chinese Biomedical Literature (CBM), to find the target researches. The random effects model was used to calculate the overall effect.

**Results:**

14 studies with 1983 participants were included, among which 13 studies were about VEGF and 1 was VEGF-B, so only the effects of VEGF were pooled. The result showed that there were obviously increased VEGF levels in DPN patients compared with diabetic patients without DPN (SMD:2.12[1.34, 2.90], *p*<0.00001) and healthy people (SMD:3.50[2.24, 4.75], *p*<0.00001). In addition, increased circulating VEGF levels were not associated with an increased risk of DPN (OR:1.02[0.99, 1.05], *p*<0.00001).

**Conclusion:**

Compared with healthy people and diabetic patients without DPN, VEGF content in the peripheral blood of DPN patients is increased, but current evidence does not support the correlation between VEGF levels and the risk of DPN. This suggests that VEGF may play a role in the pathogenesis and repairment of DPN.

## 1 Introduction

Diabetic peripheral neuropathy (DPN) is a common complication of diabetes, more than 50% of diabetic patients suffer from it, and it is a leading cause of diabetic foot ulcers and non-traumatic lower limb amputations (1). The WHO estimates that 366 million people will be affected by this disease by 2030 (2). The main clinical manifestations of DPN are lower extremity pain, numbness, paresthesia, and weakness, patients are prone to falls, ulcers, and amputations in severe cases (3). The occurrence of DPN increases the suffering of patients, reduces the quality of life, all of which aggravate the burden on families and society.

Despite the great harm and large number of patients, the pathogenesis of DPN has not been clearly explained. The occurrence of DPN is related to many pathogenic factors, including microvascular dysfunction, accumulation of advanced glycation end products(AGEs), inflammation, oxidative stress and autoimmunity (4). DPN has long been considered as a microvascular complication of diabetes, just like nephropathy and retinopathy. Clinical studies and animal experiments have confirmed that microvascular dysfunction is closely related to DPN, and the degree of microvascular injury is related to the severity of clinical symptoms (5, 6). Cumulative evidence indicates that the DPN process is accompanied by significant nerve ischemia and hypoxia (4, 7). Nerve biopsies revealed capillary basement membrane thickening, loss of capillary pericyte coverage, and endothelial hyperplasia in endoneurial microvessels (8). The series of pathological changes led to a decrease in nerve stem perfusion, resulting in the simultaneous reduction of nerve conduction velocity and oxygen tension. Angiogenesis is an important compensatory step in the body’s response to hypoxia, it can induce neovascularization to reconstruct the vascular network and restore tissue blood supply (9). Intuitively, the researchers hope to improve nerve blood supply through angiogenesis caused by tissue hypoxia, but the role of angiogenesis in DPN has been somewhat contradictory, which is partly reflected in the unclear changes of vascular endothelial growth factor (VEGF), the most representative angiogenesis factor, in DPN (10).

An important family of growth factors is vascular endothelial growth factor (VEGF) family, whose expression is mainly regulated by hypoxia-inducible factor (HIF) during hypoxia and are important participants in the angiogenesis process (11). VEGF family proteins include VEGF-A, VEGF-B, VEGF-C, VEGF-D, and placental growth factor (PLGF). Given the dominant role that VEGF-A plays in regulating angiogenesis and disease, it is also directly called VEGF (this abbreviation is used below) and will largely be the focus of this review (12). The physiological effects of VEGF are far more than angiogenesis. In recent years, VEGF has been found to have neuroprotective and nutritive effects, and is an important signaling molecule for nerve repair and regeneration (13, 14).

As an important angiogenesis and neuroprotective factor, the changes in the VEGF family in diabetic complications have attracted wide attention. However, the changes of VEGF, the most famous molecule of the VEGF family, in the peripheral circulation of DPN patients are still contradictory, and different studies have shown different trends. It has been reported that VEGF levels in the peripheral blood of DPN patients are significantly higher than those of diabetic patients without DPN and healthy people, but some studies have reached different conclusions (15, 16). Due to the current inconclusive results, we conducted this meta-analysis to comprehensively evaluate the relationship between VEGF levels in blood and DPN. For the comprehensiveness of the study, other factors in the VEGF family, such as VEGF-B, VEGF-C, VEGF-D and PLGF were also retrieved for analysis.

## 2 Methods

This study was performed according to the Preferred Reporting Items for Systematic Reviews and Meta-Analyses (PRISMA) standard and the Meta-analysis of Observational Studies in Epidemiology (MOOSE) guidelines for systematic reviews of observational studies. This study was registered at the International Prospective Register of Systematic Reviews (PROSPERO), Number CRD42023389678.

### 2.1 Literature search

Two investigators (Rui Ding and Shicong Zhu) independently conducted a systematic search on the databases of PubMed, Embase, Cochrane Library, China National Knowledge Infrastructure (CNKI), VIP Database, WanFang Database, and Chinese Biomedical Literature (CBM) through December 30, 2022, with language restrictions to Chinese and English. MeSH and free-text words are used for searching, with appropriate modifications for different databases. The search terms were used as follows: (Diabetic Neuropathy OR Diabetic Autonomic Neuropathy OR Diabetic Neuralgias OR Painful Diabetic Neuropathy OR Diabetic Asymmetric Polyneuropathy OR Diabetic Mononeuropathy OR Diabetic Polyneuropathy OR Diabetic Peripheral Neuropathy) AND (Vascular Endothelial Growth Factors OR Vascular Endothelial Growth Factor A OR VEGF-A OR Vasculotropin OR VEGF OR Vascular Permeability Factor OR Glioma-Derived Vascular Endothelial Cell Growth Factor OR GD-VEGF OR Vascular Endothelial Growth Factor B OR VEGF-B OR Vascular Endothelial Growth Factor C OR VEGF-C OR Vascular Endothelial Growth Factor D OR VEGF-D OR placental growth factor OR PLGF). More detailed search terms and procedure are in Support Material.

### 2.2 Inclusion criteria

(1) cohort, cross-sectional, or case-control studies; (2) the controls were diabetic patients without DPN or healthy controls; (3) providing mean values of VEGFs levels, or odds ratio (OR) with 95% confidence interval (95% CI) or sufficient data to calculate these levels; (4) studies in Chinese or English were considered for inclusion; If two studies used the same study population during the same period, we included only the study with larger sample size and longer follow-up.

### 2.3 Exclusion criteria

(1) studies without available data for meta-analysis were excluded; (2) reported data were on only one group of subjects; (4) obviously irrelevant studies, articles without full text, conference abstracts, comments, reviews, case-only studies, and studies based on animal models or cell lines were excluded.

### 2.4 Data extraction and quality assessment

The risk of bias was evaluated using the Newcastle Ottawa scale (NOS) for case–control studies and an adapted form of the Newcastle Ottawa scale for cross-sectional studies (17, 18). The following data were extracted from each study by 2 independent reviewers (Shicong Zhu, Xiaoyan Zhao): first author’s name, year of publication, location of the study (country), study design, study index (VEGFs family member), sample size, participants’ characteristics, mean values of VEGFs levels, OR with 95%CI on the association of VEGFs with DPN, and adjusted confounding factors.

### 2.5 Statistical analyses

All meta-analyses were performed using RevMan software (version 5.3). Data will be analyzed using a random or fixed effects model (depending on which is appropriate). Continuous variables, presented as mean ± standard deviation, with SMD and 95%CI for effective measures. The pooled OR with 95% CI was calculated for the assessment of the association between VEGFs levels and DPN risk. The *I* ^2^ index and Cochran’s Q statistics were used for heterogeneity assessment. If *p*_Q-text_< 0.1 and *I* ^2^>50%, it indicates that there is statistical heterogeneity. A sensitivity analysis to test the robustness of pooled estimates by excluding every single study was also performed. In addition, we planned a priori subgroup analysis based on: study location, study design, sample size, sample type, disease type and ages. Publication bias was assessed using funnel plot, Egger’s test and Begg’s test performed by Stata 15.0 software. If publication bias exists, we would use the trim and fill method to add several possible missing studies and recalculate the pooled estimates. Statistical significance was considered as *p* < 0.05.

## 3 Result

### 3.1 Search result

**Figure 1:**
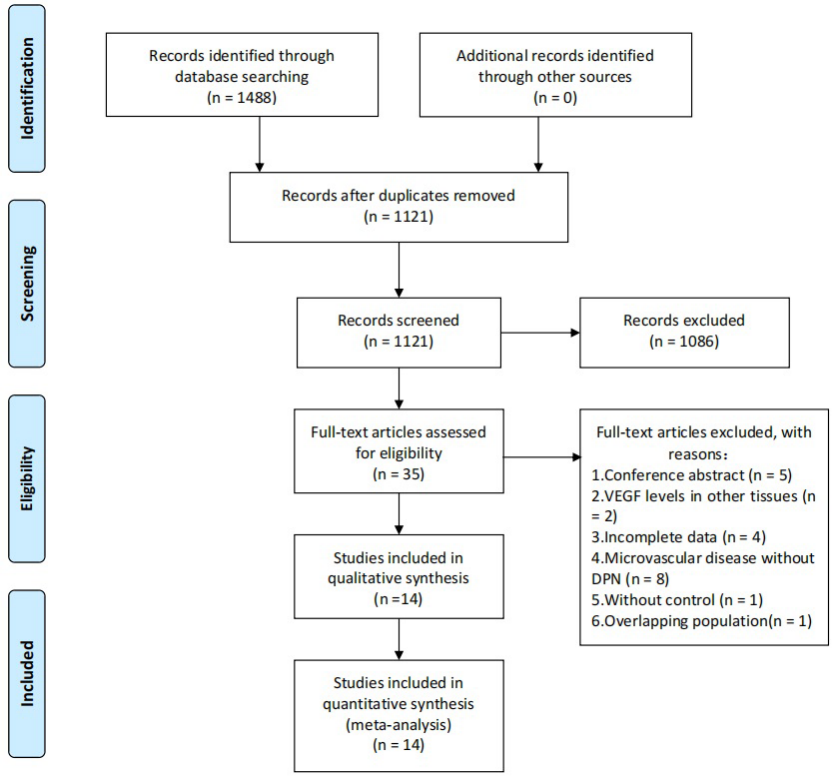
Study flow chart.

Of 1488 records (346 from CBM, 95 from CNKI, 52 from Cochrane Library, 422 from Embase, 347 from Pubmed, 44 from VIP and 182 from Wanfang) were initially identified from the online databases. After removing 367 duplicates, studies that did not meet the inclusion criteria were excluded by reading the title and abstract. After an initial review, 35 studies will be read in full text to determine whether they meet the inclusion criteria. Finally, 14 studies (19–32) were included in our meta-analysis, including 7 studies (20, 23, 24, 26, 28, 31, 32) in Chinese and 7 studies (18, 20, 21, 24, 26, 28, 29) in English. The search process is shown in **Figure 1**.

Those 14 studies involved a total of 1983 participants, including 807 DPN patients, 808 diabetic patients without DPN and 368 healthy people. Among the 14 studies, 13 studies (19–23, 25–32) reported VEGF levels in peripheral blood and 1 study (24) examined VEGF-B levels in serum. 9 (20, 22, 23, 26, 27, 29–32) studies reported circulating VEGF levels in 3 groups (healthy group, diabetic patients without DPN, DPN patients), 5 (19, 21, 24, 25, 28) studies reported VEGF levels in DPN and diabetic patients without DPN. Studies were published in 2009~2020 and conducted in 7 different countries (China, Japan, Indonesia, Iraq, Greece, Poland and Egypt). 1 study (27) specifically targeted type 1 diabetes patients, 11 studies (19, 20, 23–26, 28–32)only included type 2 diabetes patients, and the remaining 2 studies (21, 22) included both type 1 and type 2 diabetes patients. 2 studies (21, 25) graded DPN patients for severity (0~3 level) and 1 study (22) divided DPN patients into painful and non-painful DPN. 3 studies (19, 25, 31) reported the OR value between blood VEGF levels and DPN risk. 12 studies (19–25, 27, 28, 30–32) were cross-sectional studies and 2 (26, 29) were case-control studies. As to the quality, the scores of included studies ranged from 5 to 7, and the average score for the quality assessment was 6.29 (**Table 1**).

**Table 1:** Characteristics of included studies.

Author Year	Country	Study design	Groups	Sex(M/F)	Index	Sample type	Detection method	Outcomes	NOS score
Barus 2018	Indonesia	Cross-sectional	69 DPN patients 83 N-DPN patients	54/98	VEGF	Plasma	Elisa	OR	6
Cui 2012	China	Cross-sectional	33 healthy people 35 DPN patients 35 N-DPN patients	47/56	VEGF	Serum	Elisa	Mean ± SD	7
Deguchi 2009	Japan	Cross-sectional	107 DPN patients 113 N-DPN patients	105/115	VEGF	Serum	Elisa	Mean ± SD	6
Doupis 2009	Greece	Cross-sectional	50 healthy people 80 DPN patients 77 N-DPN patients	125/82	VEGF	Serum	Not Know	median (first–third quartiles)	7
Ha 2017	China	Cross-sectional	40 healthy people 35 DPN patients 35 N-DPN patients	Not Know	VEGF	Serum	Elisa	Mean ± SD	6
Hou 2022	China	Cross-sectional	38 DPN patients 65 N-DPN patients	62/41	VEGF-B	Serum	Elisa	Mean ± SD	7
Hu 2017	China	Cross-sectional	94 DPN patients 133 N-DPN patients	140/87	VEGF	Not Know	Elisa	Mean ± SD, OR	7
Huang 2015	China	Case-control	60 healthy people 60 DPN patients 60 N-DPN patients	101/79	VEGF	Serum	Elisa	Mean ± SD	5
Kuryliszyn-Moskal 2017	Poland	Cross-sectional	40 healthy people 41 DPN patients 65 N-DPN patients	49/107	VEGF	Serum	Elisa	Mean ± SD	6
Liu 2009	China	Cross-sectional	30 DPN patients 31 N-DPN patients	36/25	VEGF	Serum	Elisa	Mean ± SD	7
Mohamed 2019	Iraq	Case-control	30 healthy people 30 DPN patients 30 N-DPN patients	Not Know	VEGF	Serum	Elisa	Mean ± SD	5
Motawi 2013	Egypt	Cross-sectional	20 healthy people 40 DPN patients 20 N-DPN patients	53/27	VEGF	Plasma	Elisa	Mean ± SD	7
Tang 2020	China	Cross-sectional	40 healthy people 55 DPN patients 53 N-DPN patients	80/78	VEGF	Serum	Elisa	Mean ± SD, OR	7
Wang 2016	China	Cross-sectional	50 healthy people 51 DPN patients 50 N-DPN patients	79/72	VEGF	Plasma	Elisa	Mean ± SD	5

### 3.2 VEGF levels in diabetic patients with DPN and without DPN

**Figure 2:**
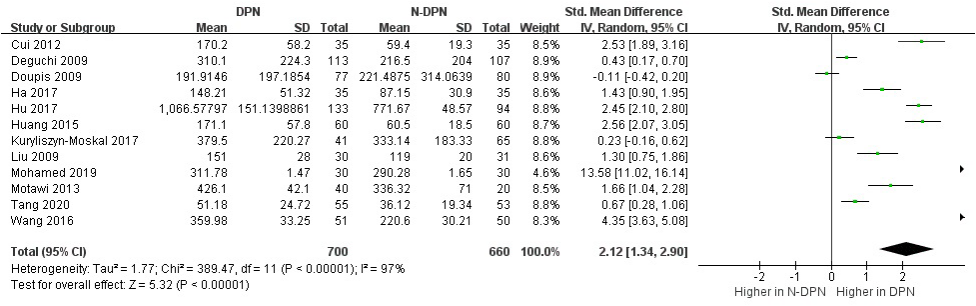
Increased VEGF levels in DPN compared with N-DPN.

**Figure 3:**
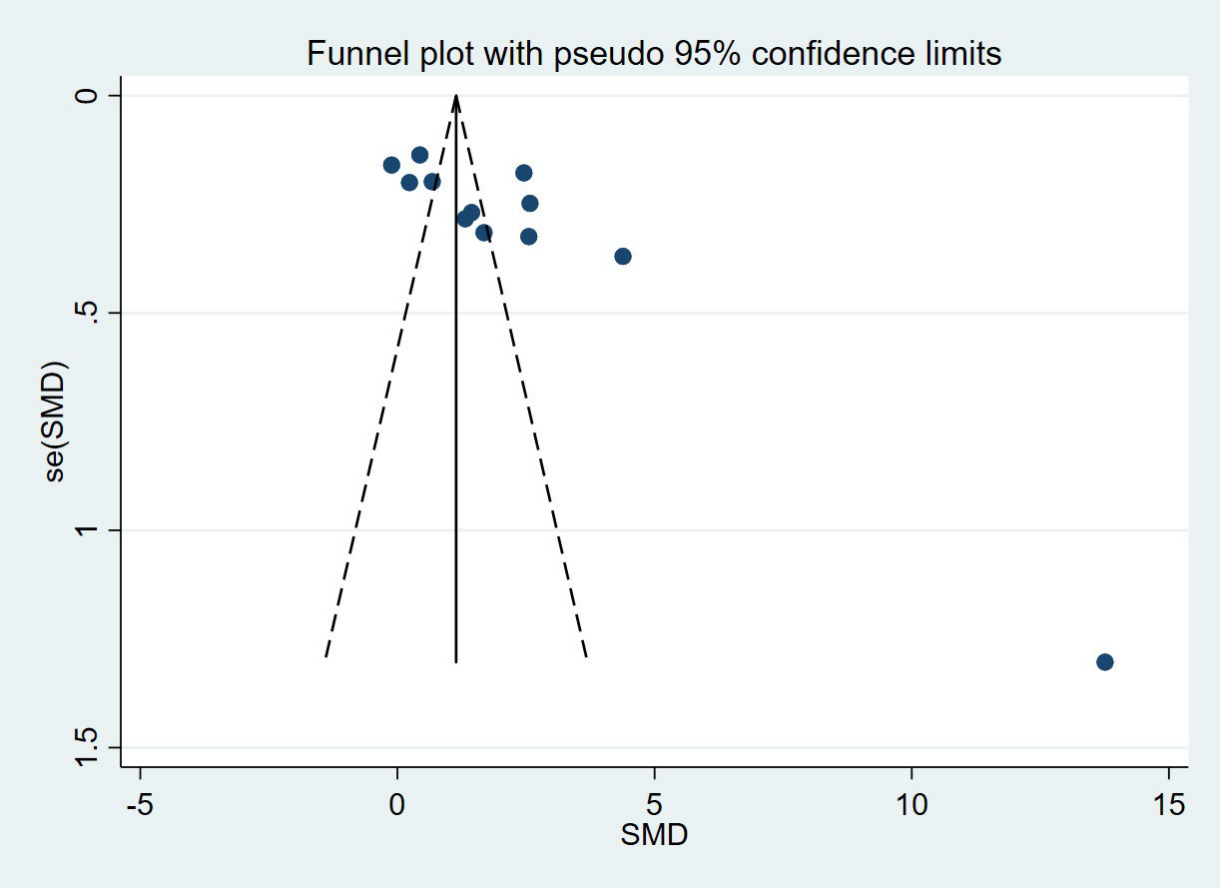
Funnel plot (DPN VS N-DPN).

**Figure 4:**
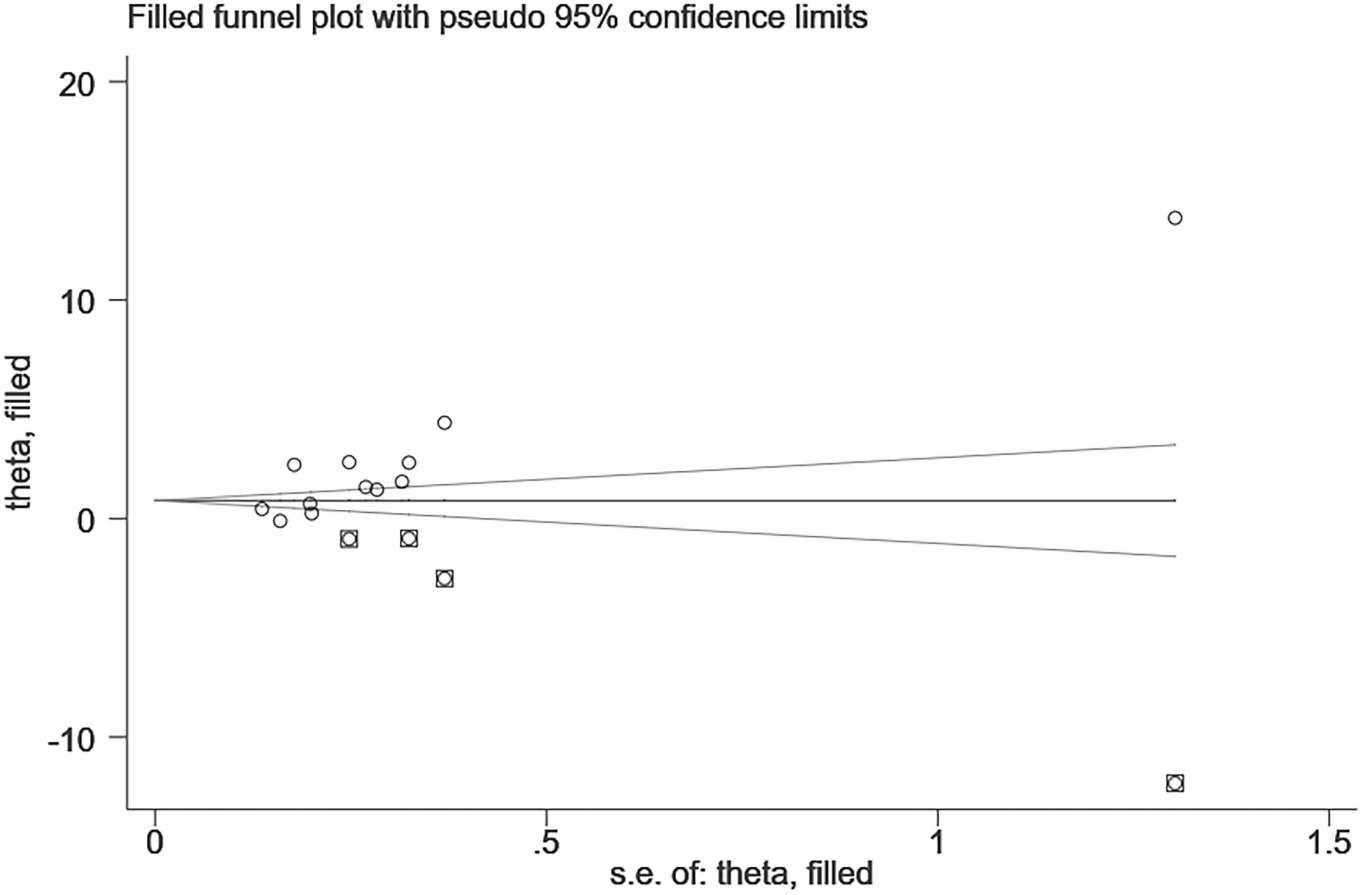
Trim and fill analysis (DPN VS N-DPN).

13 studies compared VEGF levels in the blood of DPN patients and diabetic patients without DPN (N-DPN), but 1 study did not specify the data type (e.g., median, quartile or maximum, minimum), so we pooled the analysis of VEGF levels in the 12 studies. 1 study divided DPN patients into three grades and separately reported the VEGF content in each grade, so we combined the data of the three levels. The random effects model was adopted to pool the data due to apparent heterogeneity. As shown in **Figure 2**, VEGF levels were significantly higher in DPN patients than in diabetic patients without DPN (SMD:2.12[1.34, 2.90], *p*<0.00001, Heterogeneity: Tau² = 1.77; Chi² =389.47, *P* < 0.00001; *I*² = 97%, **Figure 2**). Sensitivity analyses were performed by leaving each study separately. In this meta-analysis, sensitivity analysis did not change the high heterogeneity and the pooled estimates, which proves the stability of the present results. Draw the funnel plot of these 12 studies, and the Egger’s and Begg’s tests were conducted by Stata software to assess publication bias. The results indicated that the publication bias does exist (*p_Egger_* =0.004), the funnel plot is shown in **Figure 3**. After the trim and fill analysis imputed 4 potentially missed studies (**Figure 4**), the pooled effect (2.515[95%CI:1.089, 5.807]) is still consistent with the outcomes before the trim and fill analysis. This suggests that the result remains stable despite publication bias.

### 3.3 VEGF levels in healthy people and DPN patients

**Figure 5:**
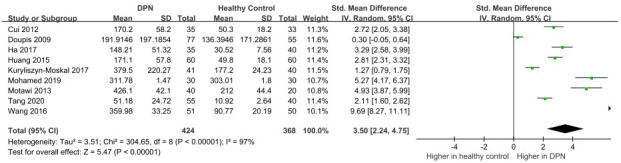
Increased VEGF levels in DPN patients compared with healthy people.

**Figure 6:**
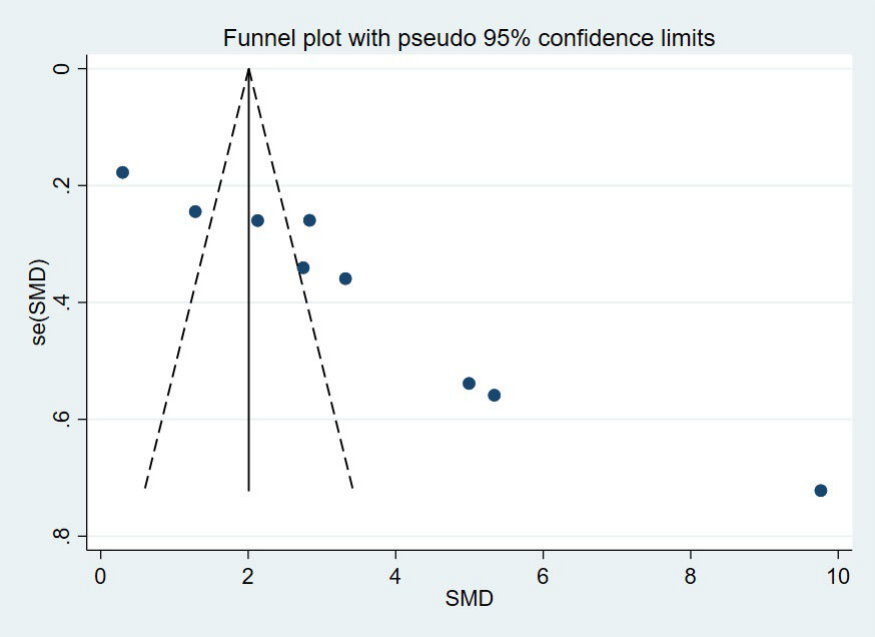
Funnel plot (DPN patients VS healthy people).

**Figure 7:**
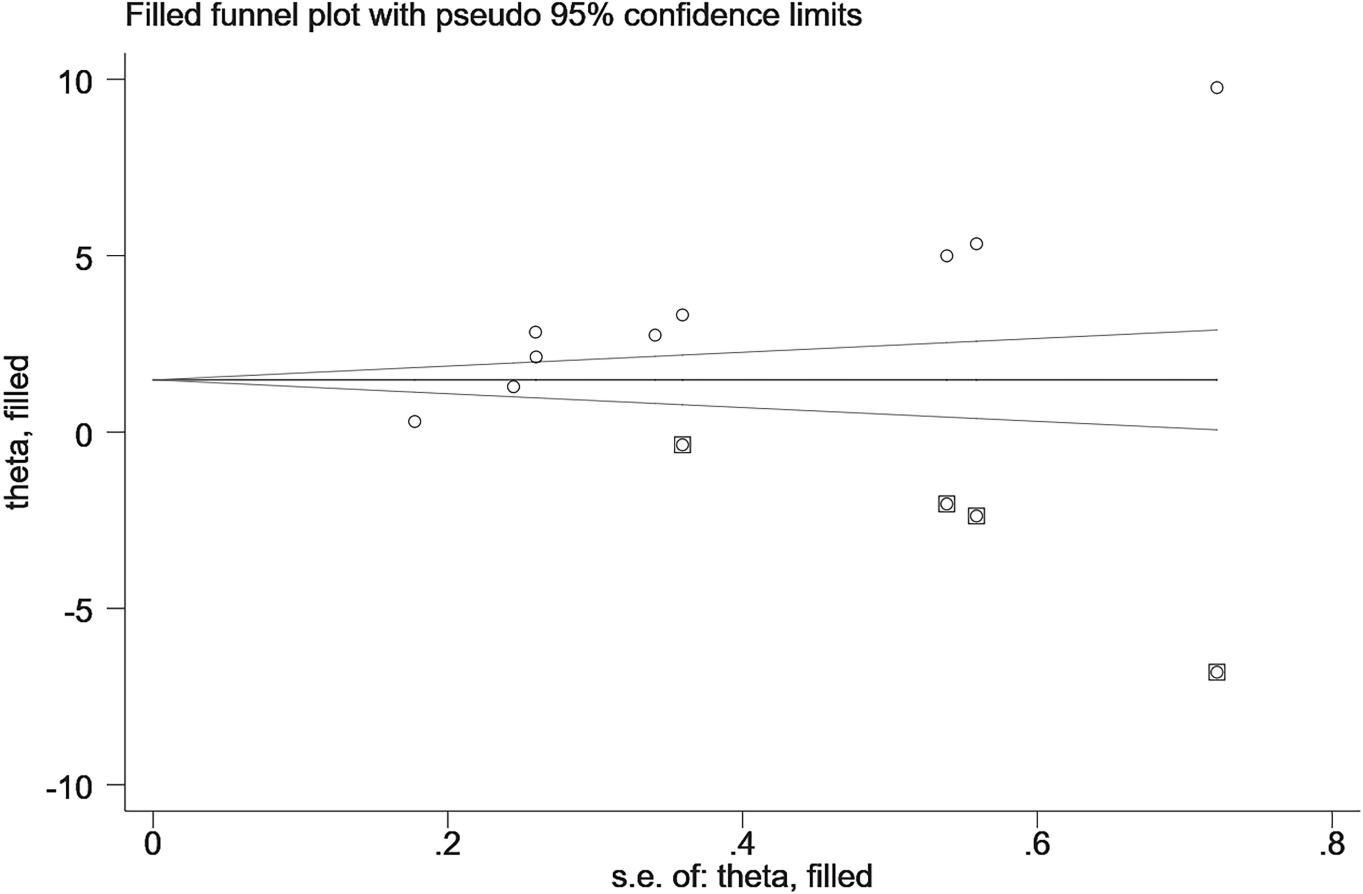
Trim and fill analysis (DPN patients VS healthy people).

There were a total of 368 healthy people from 9 studies assessing the VEGF levels compared with DPN patients. Meta-analysis of the random effect model showed that there were obviously higher VEGF levels in DPN patients compared with healthy subjects (SMD:3.50[2.24, 4.75], *p*<0.00001, Heterogeneity: Tau² = 3.51; Chi² =304.85, *P* < 0.00001; *I*² = 97%, **Figure 5**). Sensitivity analysis found no significant change in heterogeneity after excluding each study, implying that our results were stable and reliable. Considering that the source of high heterogeneity has not yet been resolved, we will then conduct a subgroup analysis to discuss the possible sources of heterogeneity according to the study location, study design, sample type, diabetes type, age, and sample size. The funnel plot of 9 studies is obviously asymmetric, and Egger’s test shows that *p_Egger_* =0.000, which indicates the existence of publication bias (**Figure 6**). Then, the trim and fill method was used to fill the possible missing studies. After adding 4 studies (**Figure 7**), the meta-analysis pooled results still strongly suggest that the VEGF levels are elevated in DPN patients compared with healthy individuals (5.083[95%CI:1.369, 18.878]).

### 3.4 Increased levels of VEGF and DPN risk

**Figure 8:**
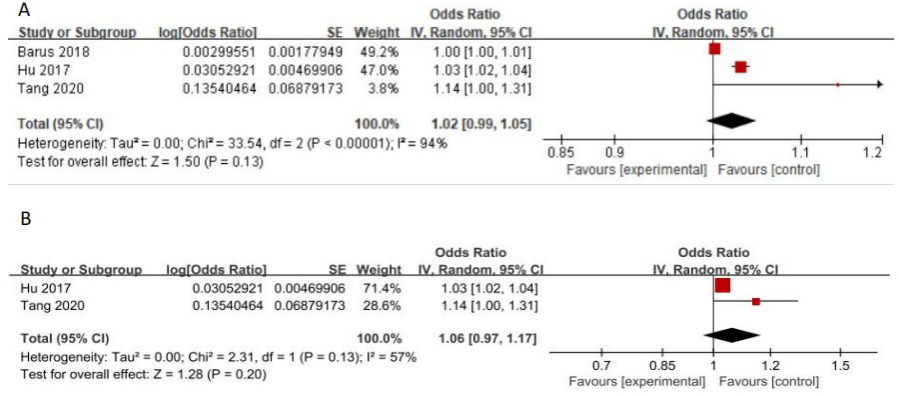
**(A)** Increased VEGF levels and the risk of DPN **(B)** Forest plot after the sensitivity analysis.

3 studies evaluated the association between elevated VEGF levels and DPN risk, while another study evaluated the association between VEGF-B and DPN. The OR values with 95%CI of 3 studies targeting VEGF were pooled for analysis. Random effects models were used to summarize the effect sizes due to high heterogeneity, and found no association between increased VEGF and DPN risk (OR:1.02[0.99, 1.05], *p*=0.13, Heterogeneity: Tau² = 0.00; Chi² =33.54, *P* < 0.00001; *I*² = 94%, **Figure 8A**). The sensitive analysis demonstrated that the article of ‘Barus 2018’ explained 37% of heterogeneity (OR:1.06[0.97, 1.17], *p*=0.20, Heterogeneity: Tau² = 0.00; Chi² =2.31, *P*=0.20; *I*² = 57%, **Figure 8B**). It can be seen that although all 3 studies independent reported that increased VEGF levels were associated with DPN risk, this association was no longer significant after our meta-analysis pooled the effect size. The article ‘hou 2018’ reported the relationship between VEGF-B level and the risk of DPN by logistic regression, which showed that VEGF-B was an independent risk factor for DPN in T2DM patients (OR: 1.441[1.154,1.797]).

### 3.5 Subgroup analysis

Since sensitivity analysis has not fully addressed the issue of high heterogeneity, we performed subgroup analysis according to different study characteristics. Subgroup analysis will be performed based on study type, study location, sample type, sample size, disease type, and age of DPN patients. The specific subgroup analysis results are shown in **Table 2**.

**Table 2:** Subgroup analysis.

	DPN VS N-DPN			DPN VS healthy people		
Characteristics	n	SMD (95% CI)	*I* ^2^	n	SMD (95% CI)	*I* ^2^
**Location**		*P=*0.71			*P*=0.39	
China	7	2.16[1.37, 2.96]	95	5	3.97[2.49, 5.44]	96
Others	5	1.90[0.78, 3.02]	97	4	2.87[0.89, 4.86]	98
**Disease type**		*P*<0.00001			*P*<0.0001	
T2DM only	9	2.81[1.91, 3.70]	96	7	4.28[3.01, 5.56]	95
T1DM or both	3	0.19[-0.15, 0.53]	70	2	0.77[-0.19, 1.72]	90
**Sample type**		*P*=0.39			*P*=0.05	
Plasma	2	3.00[0.36, 5.64]	97	2	2.48[1.43, 3.53]	96
serum	9	1.79[0.99, 2.60]	97	7	7.29[2.62, 11.95]	96
**Sample size**		*P*=0.05			–	
n≥200	3	0.92[-0.49, 2.33]	98	1	–	–
n<200	9	2.62[1.62, 3.63]	97	8	3.89[2.69, 5.10]	96
**Age**		*P*=0.26			*P*=0.26	
≥60	4	1.69[0.52, 2.86]	96	2	2.78[0.89, 4.86]	0
<60	6	3.00[1.50, 4.50]	98	7	3.74[2.10, 5.38]	98
**Study design**		*P*=0.05			*P*=0.66	
Cross-sectional	10	1.47[0.75, 2.19]	99	7	3.36[1.89, 4.83]	98
Case-control	2	8.00[-2.80, 18.79]	97	2	3.99[1.59, 6.39]	94

In those studies comparing VEGF levels in diabetic patients with DPN and without DPN, the subgroup analysis yielded the following results. In these studies, whether conducted in China or other countries, VEGF level was increased in DPN patients compared with non-DPN patients with diabetes, also this increase was independent of study location, patient age and blood sample type. However, when the subjects were only type 1 diabetes or included type 1 diabetes, the increase in VEGF became less significant (SMD: 0.19[-0.15, 0.53]). At the same time, subgroup analysis also found that different study designs and sample sizes may also affect the level of VEGF. When the number of participants ≥200, VEGF in the peripheral blood of DPN patients did not significantly increase (SMD: 0.92[-0.49, 2.33]), and the same result was also found in the pooled effect of two case-control studies (SMD: 8.00[-2.80, 18.79]). Unfortunately, the above subgroup analysis fails to account for the high heterogeneity of the study. Some possible causes of heterogeneity such as disease course, diagnostic criteria, gender, and detection methods could not be further discussed in subgroup analysis due to the incomplete and inconsistent research reports.

Our subgroup analysis of 9 studies with healthy people as controls found that different types of diabetes also affected the results of the pooled analysis, when the subjects included or only included type 1 diabetes patients, the increase of VEGF level became less significant (SMD: 0.77[-0.19, 1.72]). Simultaneously, subgroup analysis showed that there was no significant difference in subgroup according to location (*P* = 0.39), sample type (*p*=0.05), age(*p*=0.26) and study design (*P* = 0.66).

## 4 Discussion

To our knowledge, this is the first meta-analysis to evaluate the association between VEGF levels and DPN. VEGF is the most important and concerned component in the VEGF family, was also the absolute focus of the studies included in this meta-analysis. Therefore, our discussion will also focus on VEGF. Both quantitative and qualitative data of our study indicate that VEGF is overexpressed in DPN, whether compared with normal diabetic patients without DPN or healthy people. This finding is not surprising, as ischemia and hypoxia have been observed in DPN, which are the most effective stimuli to induce VEGF secretion. VEGF is known for its powerful angiogenesis effects, forming a network of blood vessels to improve tissue blood supply in response to hypoxia. Despite significant increases in VEGF levels in DPN, our study did not find an association between VEGF levels and DPN risk. Thus, it can be seen that with VEGF as the response mode, angiogenesis is involved in the pathogenesis of DPN, but VEGF as the biomarker of DPN should be treated with caution. Evidence for this conclusion was provided by a prospective study that included 315 patients with diabetes at baseline. After 5 years of follow-up, 163 patients developed DPN and 152 did not, when baseline data were compared based on DPN status, no significant differences were observed between the DPN group and non-DPN group in VEGF (33). This highlights that the effect of VEGF on DPN development is uncertain, and more prospective studies should be conducted to clarify this issue. Therefore, VEGF levels cannot be used as biomarkers for predicting DPN at the moment.

Although the subgroup analysis failed to explain the source of the heterogeneity, we were surprised to find that the increase in VEGF was no longer significant when the study population included type 1 diabetes regardless of the subjects with which DPN patients were compared (healthy people or diabetic people without DPN). This reminds us to pay attention to the role of diabetic types. Microvascular reactivity does not appear to be the same between type 1 diabetes and type 2 diabetes. VEGF has a strong ability to promote vascular permeability (34), but this permeability shows different responses in different types of diabetes. When sodium fluorescein was injected into both type 1 and type 2 diabetics, the fluorescence intensity in type 1 diabetic patients became significant 30 minutes after injection of dye, while in type 2 diabetic patients, the difference was significant only 1 minute after injection (35).The different vascular permeability and reactivity in type 1 and type 2 diabetes patients mean that different types of diabetes may have various vascular lesion characteristics and discrepant responses to VEGF. Since different types of diabetes have inconsistent responses to the same molecule, these may become potential confounding factors, leading to the emergence of heterogeneity.

Though our subgroup analysis does not find any difference this time, the content of VEGF in serum and plasma may indeed differ. Hanefeld et al. (36) found that when serum was used to detect the circulating VEGF, the serum VEGF content of T2DM patients was significantly higher than the healthy controls. However, when plasma was used, the increase became no longer significant, and the serum VEGF level was 7.3 times higher than the plasma VEGF level. VEGF is released by activated platelets, platelet activation during blood collection may also be an artificial source of VEGF, resulting in large variations in VEGF concentrations, but this is usually ignored (37). A cross-sectional study of 21 healthy subjects and 64 patients with type 1 diabetes determined VEGF levels in plasma collected in both citrate and PECT (a medium that inactivates platelets) found that higher levels of VEGF in citrate plasma samples of diabetic patients do not represent the real situation *in vivo* but mainly originate from higher artificial *ex vivo* release from platelets (38). A recent meta-analysis showed that out of the different plasma types, using EDTA as an anticoagulant obtained the highest concentration of VEGF, followed by heparin and citrate, while CTAD reported the lowest content of VEGF (39). In addition, kits from different manufacturers bring different methods for the measurement of VEGF, and other factors, including batch, sensitivity and detection range, all contribute to the study of heterogeneity. Unfortunately, due to the imperfect report, we cannot make a corresponding subgroup analysis. The above analysis reveals that the standardization problem exists in relevant measurements. Only by establishing standardization of measurement methods can future research be less heterogeneous and more comparable across studies (40). Last but not least, the pooled effect of two case-control studies suggests that VEGF levels are still higher in DPN patients than in healthy individuals, but are no longer significant when compared to diabetic patients without DPN. These 2 studies, one from China and the other from Iraq, were of low quality and did not have clear diagnostic criteria for DPN. Considering that different races, living environments, case selection bias and laboratory conditions may affect the experimental results, we should be cautious about the results of this subgroup analysis and include more high-quality studies in the future to further discuss.

DPN is the most common diabetic microvascular complication. Long-term chronic hyperglycemia leads to neuronal damage and reduced nerve blood flow, in the meantime, ischemia and hypoxia caused by neurovascular defects can also lead to structural and functional changes in nerve tissue (41, 42). VEGF enhances nerve blood flow and oxygen supply by inducing angiogenesis, in response to nerve ischemia and hypoxia caused by DPN (43). Therefore, the enhanced secretion of VEGF in DPN patients can be considered as a protective mechanism against further damage (44). VEGF levels were significantly associated with blood glucose control, as represented by HbA1c. The researchers found that plasma VEGF levels were positively correlated with FBG and HbA1c, the multiple regression analysis showed that the HbA1c level were independent predictors of VEGF levels in T2DM (45). A few studies found that functional alteration of peripheral nerves causes up-regulation of VEGF in diabetic rats, and VEGF decreased significantly under treatment (46, 47). It seems likely that VEGF levels increased as a neuroprotection strategy in response to various insults in DPN, and the decrease of VEGF may be due to the repair of the nerve after proper treatment. Indeed, some studies have found that by promoting the secretion of VEGF, the blood flow and nerve conduction velocity of the DPN sciatic nerve can be restored (48, 49). A randomized controlled trial conducted by Ropper et al. (50)found that intradermal injection of VEGF plasmid for DPN significantly reduced lower extremity symptoms in patients. Given that VEGF supplementation may have the risk of exacerbating diabetic retinopathy and inducing tumors, the study confirmed that there was no progression of diabetic retinopathy and no neoplastic disease after a year of follow-up, which preliminarily suggesting that local VEGF supplementation does not alter systemic angiogenesis status. However, several adverse events, including limb pain, edema, and muscle spasms, were reported in this study. Therefore, although VEGF supplements have been proven effective, there is still a long way to go in clinical practice due to their excessive negative effects, and more safe methods need to be found.

In diabetes mellitus, excessive or defective angiogenesis occurs. Excessive angiogenesis is often seen in diabetic retinopathy, while insufficient angiogenesis often impedes healing of diabetic foot ulcers. Abnormal angiogenesis in DPN has been specifically observed in some studies. Compared with healthy animals, functional blood vessels of the sciatic nerve in DPN animals have smaller density and shorter diameter, the content of VEGF, Ang-1 and other pro-angiogenesis regulatory factors in the sciatic nerve is lower (5, 51). Thus, some angiogenic factors (VEGF, Ang-1) have been proposed to treat peripheral neuropathy (52, 53). However, it must be pointed out that some studies have found that VEGF levels in the sciatic nerve increased after STZ induction (54). These findings are not contradictory, as we must consider the possible effects of the disease course. Chavez et al. (55) confirmed that the expression of HIF-1α and its downstream factors (VEGF, EPO) in diabetic sciatic nerve showed a trend of first increasing and then decreasing in chronological order. Current human studies have found a negative association between endoneurial capillary density and disturbed nerve dysfunction, suggesting a compensatory increase in vascular density early in neuropathy (56). The 2 studies (21, 25) we included divided DPN into 4 stages, among which stage 0 was no evidence of DPN, stage 1~3 was divided according to severity, stage 1 was no symptoms, but abnormal signs of DPN, and stage 3 was disabling neuropathy. In both studies, stage 3 has the longest course of the disease, but stage 2 has the largest level of VEGF, which at least partially supports the above view, that is, the level of VEGF increases first and then decreases with the course or severity of the disease. This conclusion also implies that the proposed ‘angiogenic’ strategies to treat microangiopathy in nerves might not be suitable at every stage of DPN. As for why the compensatory effect of angiogenesis is weakened with the progression of the disease, studies have shown that high glucose can damage the HIF-1α signaling pathway under hypoxia conditions and reduce the levels of VEGF, but this damage may take time to accumulate. Hyperglycemia disrupts HIF-1α stability and this disruption may be mediated by PHD or VHL, PHD inhibition or VHL inactivation can largely rescue HIF-1α stability, but the underlying mechanism is not fully understood (57). What’s more, hyperglycemia also induces methylglyoxal (MGO) accumulation, which induces the degradation of HIF-1α and decreases the transcriptional activity of HIF-1. MGO modifies HIF-1α to increase its association with heat shock protein 40/70 (Hsp40/70), leading to CHIP (Carboxy terminus of Hsp70-Interacting Protein) recruitment, thereby promoting HIF-1α ubiquitination and degradation (58). MGO also modification of P300, which inhibits its recruitment of HIF-1α, and produces a decrease in VEGF expression (59). Although high glucose inhibits the HIF-1α response and the expression of VEGF, at the same time, the continuous existence of glucose metabolism disorder in diabetes means that the hypoxia factor cannot change and still stimulates the expression of HIF-1α and VEGF. Therefore, the blood VEGF content in DPN patients is still higher than that in healthy people and diabetic patients without DPN until decompensation occurs.

The limitations of our study should be acknowledged. First, no eligible prospective cohort studies were found in our search, thus no causal relationship could be explored. Second, only one study focused exclusively on type 1 diabetes, so a more detailed analysis of diabetes types was not possible. Third, the OR values involved in this study were all unadjusted, so more adjusted OR is needed to refine the conclusion. Fourth, most of the included studies were rated as medium in quality. In view of the fact that high-quality studies can provide more effective information for analysis, more excellent trials should be added in the future. Finally, high heterogeneity and publication bias reminds us to treat the results of this study with caution.

## 5 Conclusion

In conclusion, the circulating VEGF levels in DPN patients were significantly higher than that in diabetic patients without DPN and healthy controls. This meta-analysis did not find a connection between the level of VEGF in peripheral blood and the risk of DPN. Due to the limitations of this study, more high-quality studies, especially cohort studies, are needed in the future to further clarify the relationship between DPN and VEGF. In addition, although our studies have proved that VEGF plays an important role in the pathogenesis and treatment of DPN, it still needs careful consideration as a biomarker and therapeutic approach.

## Author contributions

RD collected, analyzed, designed this study, and wrote the main manuscript as the first author. SZ and XZ extracted data and performed the statistical analysis. RD and SZ retrieved the literature and conducted a research quality assessment. RY supervised the work. All authors contributed to the article and approved the final manuscript.
