# Corrigendum: Vascular endothelial growth factor levels in diabetic peripheral neuropathy: a systematic review and meta-analysis

**DOI:** 10.3389/fendo.2023.1238758

**Published:** 2023-08-14

**Authors:** Rui Ding, Shicong Zhu, Xiaoyan Zhao, Rensong Yue

**Affiliations:** ^1^ Department of Endocrinology, Hospital of Chengdu University of Traditional Chinese Medicine, Chengdu, China; ^2^ Department of Respiratory, Hospital of Chengdu University of Traditional Chinese Medicine, Chengdu, China

**Keywords:** diabetic peripheral neuropathy, vascular endothelial growth factor, angiogenesis, microcirculation, meta analysis

In the published article, there was an error regarding the affiliation for Shicong Zhu. Instead of having affiliation 1, they should have *Department of Respiratory, Hospital of Chengdu University of Traditional Chinese Medicine, Chengdu, China*.

The authors apologize for this error and state that this does not change the scientific conclusions of the article in any way. The original article has been updated.

