# Supplementary material for: Vascular endothelial growth factor levels in diabetic peripheral neuropathy: a systematic review and meta-analysis
Source: Front Endocrinol (Lausanne). 2023 May 12;14:1169405. doi: 10.3389/fendo.2023.1169405 (PMC10213658; doi:10.3389/fendo.2023.1169405)
Supplement: Supplementary file 1 [file DataSheet_1.pdf]

Supplementary Material

Cochrane Library search records

#1 MeSH descriptor: [Vascular Endothelial Growth Factors] explode all trees

#2 (Vascular Endothelial Growth Factor-A):ab,ti,kw OR (vasculotropi A):ab,ti,kw OR (VEGF-A):ab,ti,kw OR (Vasculotropin):ab,ti,kw OR (VEGF):ab,ti,kw OR (Vascular Endothelial Growth Factor A):ab,ti,kw OR (Vascular Permeability Factor):ab,ti,kw OR (Permeability Factor, Vascular):ab,ti,kw OR (Glioma-Derived Vascular Endothelial Cell Growth Factor):ab,ti,kw OR (Glioma Derived Vascular Endothelial Cell Growth Factor):ab,ti,kw OR (GD-VEGF):ab,ti,kw OR (VEGF-B):ab,ti,kw OR (Vascular Endothelial Growth Factor C):ab,ti,kw OR (VEGF-C):ab,ti,kw OR (Vascular Endothelial Growth Factor D):ab,ti,kw OR (VEGF-D):ab,ti,kw OR (placental growth factor):ab,ti,kw OR (PLGF):ab,ti,kw

#3 (neuropathies, diabetic):ab,ti,kw OR (diabetic neuropathy):ab,ti,kw OR (Neuropathy, Diabetic):ab,ti,kw OR (Diabetic Autonomic Neuropathy):ab,ti,kw OR (Autonomic Neuropathies, Diabetic):ab,ti,kw OR (Autonomic Neuropathy, Diabetic):ab,ti,kw OR (Diabetic Autonomic Neuropathies):ab,ti,kw OR (Neuropathies, Diabetic Autonomic):ab,ti,kw OR (Neuropathy, Diabetic Autonomic):ab,ti,kw OR (Diabetic Neuralgia):ab,ti,kw OR (Diabetic Neuralgias):ab,ti,kw OR (Neuralgias, Diabetic):ab,ti,kw OR (Diabetic Neuropathy, Painful):ab,ti,kw OR (Diabetic Neuropathies, Painful):ab,ti,kw OR (Neuropathies, Painful Diabetic):ab,ti,kw OR (Neuropathy, Painful Diabetic):ab,ti,kw OR (Painful Diabetic Neuropathies):ab,ti,kw OR (Painful Diabetic Neuropathy):ab,ti,kw OR (Neuralgia, Diabetic):ab,ti,kw OR (Symmetric Diabetic Proximal Motor Neuropathy):ab,ti,kw OR (Asymmetric Diabetic Proximal Motor Neuropathy):ab,ti,kw OR (Diabetic Asymmetric Polyneuropathy):ab,ti,kw OR (Asymmetric Polyneuropathies, Diabetic):ab,ti,kw OR (Asymmetric Polyneuropathy, Diabetic):ab,ti,kw OR (Diabetic Asymmetric Polyneuropathies):ab,ti,kw OR (Polyneuropathies, Diabetic Asymmetric):ab,ti,kw OR (Polyneuropathy, Diabetic Asymmetric):ab,ti,kw OR (Diabetic Mononeuropathy):ab,ti,kw OR (Diabetic Mononeuropathies):ab,ti,kw OR (Mononeuropathies, Diabetic):ab,ti,kw OR (Mononeuropathy, Diabetic):ab,ti,kw OR (Diabetic Mononeuropathy Simplex):ab,ti,kw OR (Diabetic Mononeuropathy Simplices):ab,ti,kw OR (Mononeuropathy Simplex, Diabetic):ab,ti,kw OR (Mononeuropathy Simplices, Diabetic):ab,ti,kw OR (Simplex, Diabetic Mononeuropathy):ab,ti,kw OR (Simplices, Diabetic Mononeuropathy):ab,ti,kw OR (Diabetic Amyotrophy):ab,ti,kw OR (Amyotrophies, Diabetic):ab,ti,kw OR (Amyotrophy, Diabetic):ab,ti,kw OR (Diabetic Amyotrophies):ab,ti,kw OR (Diabetic Polyneuropathy):ab,ti,kw OR (Diabetic Polyneuropathies):ab,ti,kw OR (Polyneuropathies, Diabetic):ab,ti,kw OR (Polyneuropathy, Diabetic):ab,ti,kw

#4 MeSH descriptor: [Diabetic Neuropathies] explode all trees

#5 #1 OR #2

#6 #3 OR #4

#7 #5 AND #6

Embase search records

#1 'diabetic neuropathy'/exp

#2 'diabetic neuropathies':ab,ti OR 'neuropathies, diabetic':ab,ti OR 'neuropathy, diabetic':ab,ti OR 'diabetic autonomic neuropathy':ab,ti OR 'autonomic neuropathies, diabetic':ab,ti OR 'autonomic neuropathy, diabetic':ab,ti OR 'diabetic autonomic neuropathies':ab,ti OR 'neuropathies, diabetic autonomic':ab,ti OR 'neuropathy, diabetic autonomic':ab,ti OR 'diabetic neuralgia':ab,ti OR 'diabetic neuralgias':ab,ti OR 'neuralgias, diabetic':ab,ti OR 'diabetic neuropathy, painful':ab,ti OR 'diabetic neuropathies, painful':ab,ti OR 'neuropathies, painful diabetic':ab,ti OR 'neuropathy, painful diabetic':ab,ti OR 'painful diabetic neuropathies':ab,ti OR 'painful diabetic neuropathy':ab,ti OR 'neuralgia, diabetic':ab,ti OR 'symmetric diabetic proximal motor neuropathy':ab,ti OR 'asymmetric diabetic proximal motor neuropathy':ab,ti OR 'diabetic asymmetric polyneuropathy':ab,ti OR 'asymmetric polyneuropathies, diabetic':ab,ti OR 'asymmetric polyneuropathy, diabetic':ab,ti OR 'diabetic asymmetric polyneuropathies':ab,ti OR 'polyneuropathies, diabetic asymmetric':ab,ti OR 'polyneuropathy, diabetic asymmetric':ab,ti OR 'diabetic mononeuropathy':ab,ti OR 'diabetic mononeuropathies':ab,ti OR 'mononeuropathies, diabetic':ab,ti OR 'mononeuropathy, diabetic':ab,ti OR 'diabetic mononeuropathy simplex':ab,ti OR 'diabetic mononeuropathy simplices':ab,ti OR 'mononeuropathy simplex, diabetic':ab,ti OR 'mononeuropathy simplices, diabetic':ab,ti OR 'simplex, diabetic mononeuropathy':ab,ti OR 'simplices, diabetic mononeuropathy':ab,ti OR 'diabetic amyotrophy':ab,ti OR 'amyotrophies, diabetic':ab,ti OR 'amyotrophy, diabetic':ab,ti OR 'diabetic amyotrophies':ab,ti OR 'diabetic polyneuropathy':ab,ti OR 'diabetic polyneuropathies':ab,ti OR 'polyneuropathies, diabetic':ab,ti OR 'polyneuropathy, diabetic':ab,ti

#3 'vasculotropin'/exp

#4 'vegf-a':ab,ti OR 'vasculotropin a':ab,ti OR 'vegf':ab,ti OR 'vascular endothelial growth factor':ab,ti OR 'vascular permeability factor':ab,ti OR 'permeability factor, vascular':ab,ti OR 'glioma-derived vascular endothelial cell growth factor':ab,ti OR 'glioma derived vascular endothelial cell growth factor':ab,ti OR 'gd-vegf':ab,ti OR 'vascular endothelial growth factor b':ab,ti OR 'vegf-b':ab,ti OR 'vascular endothelial growth factor c':ab,ti OR 'vegf-c':ab,ti OR 'vascular endothelial growth factor d':ab,ti OR 'vegf-d':ab,ti OR 'placental growth factor':ab,ti OR 'plgf':ab,ti

#5 #1 OR #2

#6 #3 OR #4

#7 #5 AND #6
